# Special Issue “Targeted Therapy of Cancer: Innovative Drugs and Molecular Tools”

**DOI:** 10.3390/ijms27020657

**Published:** 2026-01-09

**Authors:** Francesco Giansanti, Rodolfo Ippoliti

**Affiliations:** Department of Life, Health and Environmental Sciences, University of L’Aquila, 67100 L’Aquila, Italy; rodolfo.ippoliti@univaq.it

Modern medicine has focused its attention on the possibility of delivering drugs in a controlled manner, particularly as soon as more attractive targets have emerged, and new molecular tools have become available. Targeted cancer therapy has become an innovative strategy, with new drugs being constantly discovered and applied in the treatment of various types of tumors, enabling precision and personalized medicine. A major challenge concerns the safe delivery of drugs (minimizing side-effects) and the potential exploitation of novel molecular tools such as miRNA, CRISPR-Cas9, aptamers, and extracellular vesicles (EVs).

These approaches complement and integrate the use of more classical methods, such as antibody-based therapies, well-established and clinically effective in the field of cancer treatment [1].

We would like to thank everyone who made the publication of this Special Issue possible: the authors, peer reviewers, and editorial team. Everyone’s work has made it possible to explore such a vast and varied field of research, ultimately providing a broad and up-to-date overview of the strategies for targeted cancer therapy including the mechanisms and optimization of Targeted Radionuclide Therapy, the identification of new therapeutic targets such as CD320, and the role of molecular regulation in improving treatment strategies. It also highlights key signaling pathways involved in cancer progression and the development of more effective CAR-T cell therapies, with the overall goal of enhancing anti-cancer efficacy and improving patient outcomes.

Together, these studies suggest innovative strategies advancing modern cancer therapy—from improving CAR-T cell manufacturing and optimizing radiotherapy effects to developing molecularly targeted, immune-modulating, and anti-metastatic approaches. They collectively reflect a shift toward personalized, mechanism-based oncology aimed at overcoming resistance and reducing systemic toxicity.

Yassin et al. reports that CAR-T cell therapy has revolutionized the treatment of refractory hematologic cancers but faces production challenges, particularly low viral transduction efficiency and T-cell variability [2]. The proposed strategy involves culturing T cells in serum-free Nutri-T medium and treating them for 24 h with low doses of BX795 and rosuvastatin [3,4]. This combination improves CAR-T transduction and functionality, offering a promising method to produce more effective cells for cancer immunotherapy.

Ferro-Flores et al. examined Targeted Radionuclide Therapy (TRT) using radiopharmaceuticals to selectively deliver radiation to tumor cells while minimizing damage to healthy tissues. Beyond direct radiation effects, TRT induces non-targeted effects—such as bystander and abscopal responses—that enhance its therapeutic potential [5]. It also modulates immune responses, boosting the efficacy of combination therapies. Despite success in cancers like prostate and neuroendocrine tumors, challenges like hypoxia and radioresistance persist [6]. Advances in theranostics, AI, and precision dosimetry are paving the way for more personalized and effective TRT approaches.

Tolymbekova et al. examined the CD320 receptor, responsible for vitamin B12 uptake, overexpressed in several cancers and representing a potential selective therapeutic target [7]. Current strategies include vitamin B12-drug conjugates, CD320-specific antibodies or nanobodies, and nanoparticles carrying cytotoxic agents [8,9,10]. CD320 may also serve as an early cancer biomarker, but further in vivo studies are needed to assess its therapeutic safety and efficacy.

Ahmetoglu et al. investigated the role of guanylate-binding proteins (GBP1–7), interferon-inducible GTPases that defend against intracellular pathogens [11]. In cancer, their roles are context-dependent—they can either promote malignancy by enhancing immune signaling and therapy resistance or suppress tumors by activating immune pathways and regulating cell proliferation. Understanding GBP regulation could open up new opportunities for prognostic use and targeted cancer therapies [12,13,14].

Farhadi et al. studied the adaptor protein p130Cas that interacts with Crk and CrkL, regulating cell adhesion, migration, and invasion—processes crucial in tumor progression and metastasis [15,16,17]. Dysregulation of this signaling axis is linked to cancer development and aggressiveness. Targeting the p130Cas–Crk/CrkL complex could therefore inhibit cancer cell dissemination and serve as a novel therapeutic approach [18].

The ultimate goal of this Special Issue was to provide further ideas for developing innovative strategies, promoting collaboration between different disciplines, and thus contributing to the development of new, more effective, and sustainable treatments.

Seven manuscripts were submitted for consideration for the Special Issue and finally five papers were accepted for publication.
